# Influence of Printing Orientation and Ageing on Mechanical Properties of 3D-Printed Resins for Occlusal Splints

**DOI:** 10.3390/ma19061079

**Published:** 2026-03-11

**Authors:** Carlo Bosoni, Alessandro Vichi, Lorenzo Franchi, Hanan Al-Johani, Cecilia Goracci

**Affiliations:** 1 Department of Experimental and Clinical Medicine, University of Florence, 50134 Florence, Italy; carlo.bosoni@unifi.it (C.B.); lorenzo.franchi@unifi.it (L.F.); 2 School of Dental, Health and Care Professions, University of Portsmouth, Portsmouth PO1 2EG, UK; alessandro.vichi@port.ac.uk; 3 Department of Restorative Dentistry, Faculty of Dentistry, King Abdulaziz University, Jeddah 21589, Saudi Arabia; 4 Department of Medical Biotechnologies, University of Siena, 53100 Siena, Italy; cecilia.goracci@unisi.it

**Keywords:** 3D printing, resin, occlusal splint, printing orientation, flexural strength, flexural modulus, water ageing

## Abstract

The aim of this study was to assess the effect of printing orientation and water ageing on the flexural strength and flexural modulus of 3D printed resins for occlusal splints. Bar-shaped specimens were designed with dimensions of 64 × 10 × 3.3 mm according to ISO 20795-2:2013. Specimens were 3D printed with the Form 3B printer (Formlabs), using Dental LT Clear Resin (CL) or Comfort Resin (CO) (Formlabs), and 3 different printing orientations: as per manufacturer’s recommendation (40° N = 20), parallel (0° N = 20), or perpendicular to the build platform (90° N = 20). To simulate intraoral ageing, half of the specimens per material and printing orientation (N = 10) were stored in distilled water at 37 °C for 30 days prior to testing. Specimens were tested in a three-point bending apparatus using a universal testing machine equipped with a 50 N load cell moving at a crosshead speed of 5 mm/min. Flexural strength (MPa) and flexural modulus (GPa) data were collected and statistically processed with separate analyses for unaged and aged specimens (Two-Way or One-Way ANOVA; Tukey test; *p* < 0.05). As for unaged specimens, both resin materials exhibited the highest flexural strength and modulus in the 90° orientation and the lowest values in the 40° orientation group. After water aging, all groups showed reduced flexural strength and modulus, with CO displaying up to 52% loss in flexural strength and values falling below ISO thresholds. CO consistently exhibited significantly lower flexural strength and modulus than CL, irrespective of aging.

## 1. Introduction

With a groundbreaking impact on dentistry, additive manufacturing, also known as 3D printing, has been effectively utilized for the fabrication of several customized dental devices, including occlusal splints [1,2]. Occlusal splints are intraoral devices commonly used to address temporomandibular joint disorders and parafunctional muscular activities by modifying occlusal contacts and jaw relationship, with the intention to reduce muscle tightness and alleviate clinical symptoms. They also provide protection against tooth wear [3,4]. Traditionally, occlusal splints have been manufactured with conventional methods, such as wax modelling and thermoforming techniques [5]. However, the new technologies of intraoral scanning, 3D modelling, and 3D printing have enabled the production of occlusal splints with enhanced fit and greater control over material thicknesses, easing the achievement of occlusal contact balance. Reduction in laboratory time and limitation of material waste are also reported benefits of the digital workflow [3,6,7]. The variety of available materials has been considered as another advantage of 3D printing in dentistry, and resins specifically meant for occlusal splint fabrication have been marketed [2].

When assessing the mechanical properties of 3D-printed occlusal splints [7,8], several factors, such as resin composition [9,10,11,12,13], printing technology [13,14], layer thickness [14,15,16], and post-curing methods [15,16,17] were found to be relevant. Nevertheless, printing orientation [16,18,19,20,21,22,23,24,25] and storage media [11,13,15,23,26,27,28,29] were identified as the most influential variables.

As 3D printing produces the manufacturing in a layer-wise manner, printing orientation has been reported to have a significant influence on the flexural strength and modulus of 3D-printed devices [18,30]. When a force is applied, the between-layer interfaces are deemed as points of weakness, owing to the possible presence of voids or to insufficient adhesion between the layers [31]. Moreover, the layered configuration renders the 3D printed structures inherently anisotropic, i.e., displaying different mechanical behavior depending on the direction of the force applied. Anisotropy, in turn, generates varying levels of internal stresses within the printed devices, which can impact their resistance to bending [18,30]. It therefore appeared interesting to verify to what extent the printing orientation parameter affects the mechanical properties of resins indicated for 3D printing of occlusal splints [19,30].

Another phenomenon with relevant influence on the mechanical characteristics of dental resins is water sorption, wherein resin materials absorb moisture from the surroundings. Prolonged water sorption induces the hydrolytic degradation of the chemical bonds between the resin matrix and surrounding fillers, and triggers the release of leachable resin elements into the oral environment, both of which cumulatively weaken the integrity of the overall 3D printed polymer structure [32]. Water sorption of 3D-printed resin restorations has been confirmed to adversely impact their dimensional accuracy, mechanical durability, and long-term stability by means of the plasticizing effect of water molecules [30,32,33,34,35].

Few studies previously assessed the mechanical properties of 3D printed resins for occlusal splints after water storage meant to simulate intraoral ageing [10,13,15]. However, the adopted experimental set-ups differed largely across studies, hindering a meaningful comparison of their findings. Additionally, the current literature has overlooked a 3D printed resin that has gained significant diffusion in the dental community, also due to its use in the fabrication of orthodontic retainers. The referred resin is Dental LT Clear v2 (Formlabs, Sommerville, MA, USA). Lately, the same manufacturer has introduced an innovative resin for 3D printing of occlusal splints, Dental LT Comfort (Formlabs), stating that the material provides increased flexibility, greater resistance to fracture and wear, in addition to enhanced transparency. These claims, however, need to be validated independently.

Thereby, the present study was aimed at comparatively assessing the mechanical behavior under flexural loading of Dental LT Clear v2 and Comfort resin specimens, printed at different angulations to the build platform, before and after water storage. Flexural strength and flexural modulus data were acquired using a three-point bending model. The tested null hypothesis was that no difference in flexural strength or modulus existed between the two materials, regardless of printing orientation and ageing.

## 2. Materials and Methods

### 2.1. Specimen Preparation

Table 1 reports the chemical composition of the two tested resins: Dental LT Clear v2 (CL) and Dental LT Comfort (CO).

Following ISO 20795-2:2013 [36], which applies to orthodontic base polymers and copolymers used in both active and passive orthodontic appliances, specimens with dimensions of 64 × 10 × 3.3 mm were designed using Tinkercad software (https://www.tinkercad.com, accessed online on 3 March 2023) (Autodesk, San Rafael, CA, USA). The design file was exported in STL format and imported into PreForm software 3.28.1 (Formlabs, Somerville, MA, USA) for automatic support generation and slicing.

**Figure 1:**
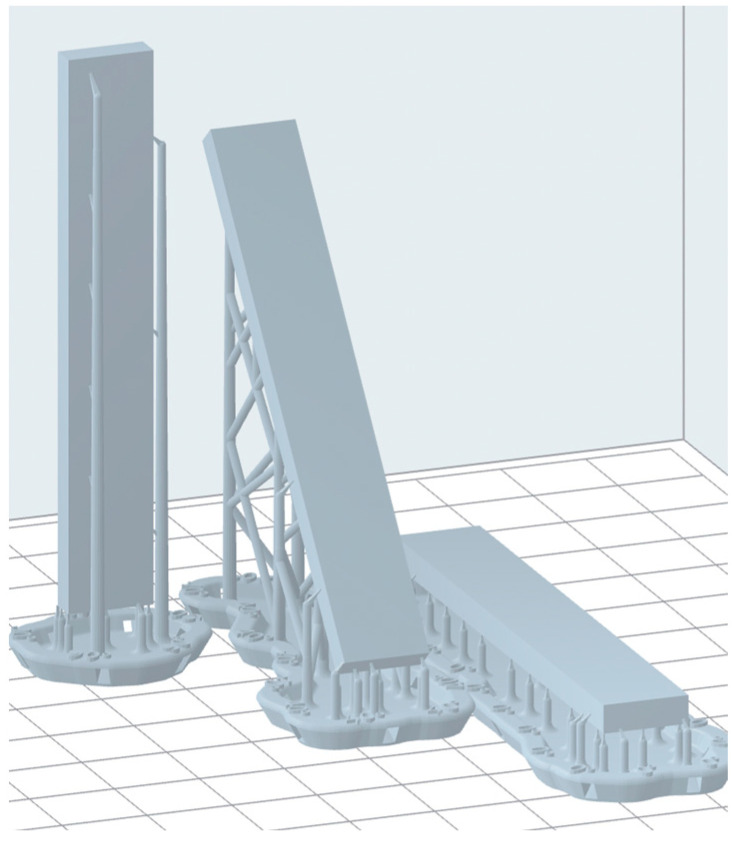
Digital diagram showing the different printing orientations (90°, 40°, 0°) for the resin specimens.

To assess the influence of printing orientation on flexural properties, specimens were designed at three different orientations relative to the build platform: parallel (0°), perpendicular (90°), and manufacturer-recommended angulation (40°) (Figure 1).

For each material and printing orientation, 20 specimens were fabricated using a Form 3B stereolithography (SLA) printer (Formlabs, Somerville, MA, USA), with a layer thickness of 100 µm. CL specimens were washed in isopropyl alcohol (IPA, ≥99%) using the Form Wash device (Formlabs, Somerville, MA, USA) for 15 min. An additional 5 min wash in fresh IPA followed. After drying at room temperature for at least 30 min, specimens were post-cured in the Form Cure curing machine (Formlabs, Somerville, MA, USA) at 60 °C for 60 min, according to the manufacturer’s recommendations. CO specimens were washed with IPA (≥99%) in the Form Wash device (Formlabs, Somerville, MA, USA) for 10 min. After drying at room temperature for at least 30 min, specimens were post-cured in the Form Cure machine (Formlabs, Somerville, MA, USA) at 60 °C for 20 min, as per the manufacturer’s instructions. Subsequently, supports were removed using a cutting disk (Horico Diamond Disc Double Sided Handpiece 355C/220 2.2 mm, HORICO DENTAL Hopf, Ringleb & Co. GmbH & Cie, Berlin, Germany), mounted on a handpiece.

In accordance with ISO 20795-2:2013 [36], the height of the specimens was measured at three points along the long axis using a digital caliper (Beta Utensili S.p.A., Sovico, Italy) with an accuracy of ±0.01 mm, and it was verified that the deviation between measurements did not exceed ±0.02 mm.

### 2.2. Artificial Ageing Protocol

Still in accordance with ISO 20795-2:2013 [36], all the specimens were stored in a water bath at 37 °C for 5 min to simulate the oral environment [37]. For each combination of material and printing orientation, half of the specimens, selected at random, were tested immediately after, at room temperature, and under dry conditions. The remaining specimens were stored in distilled water at 37 °C for 30 days prior to testing.

### 2.3. Flexural Strength Test

**Figure 2:**
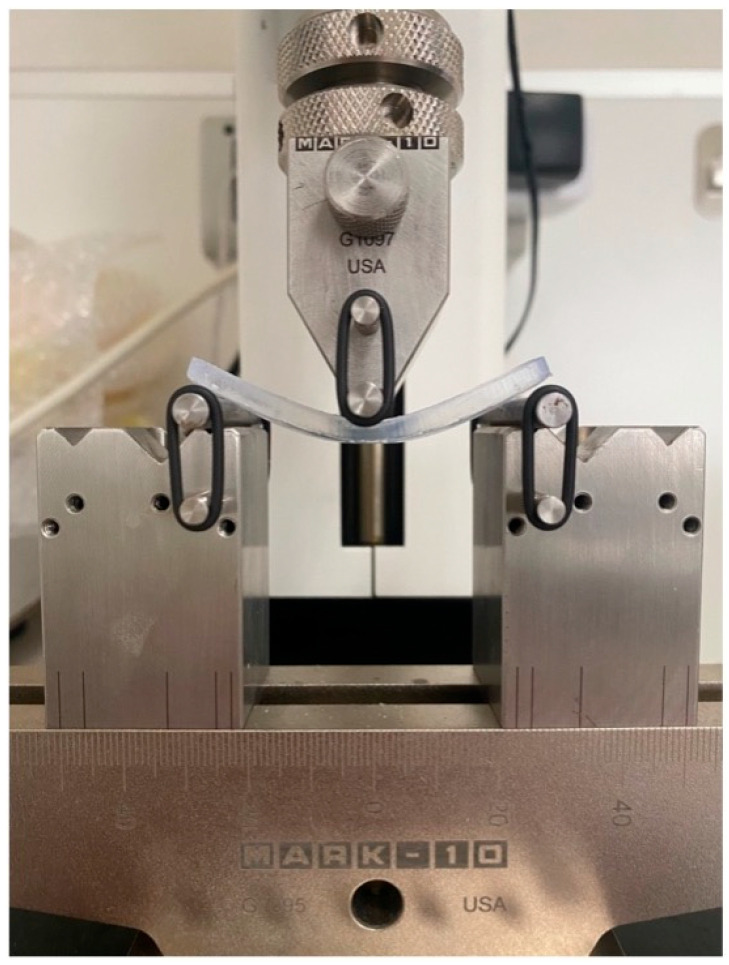
Experimental setup for the three-point bending test. The resin specimen is positioned with its central portion aligned under the crosshead.

The three-point bending test apparatus consisted of a central loading plunger (anvil) and two cylindrical supports (rollers), each 3.2 mm in diameter. The distance between the centers of the supports was maintained at 50 ± 0.1 mm, and the loading plunger was positioned within 0.1 mm of the midpoint between the supports (Figure 2).

**Figure 3:**
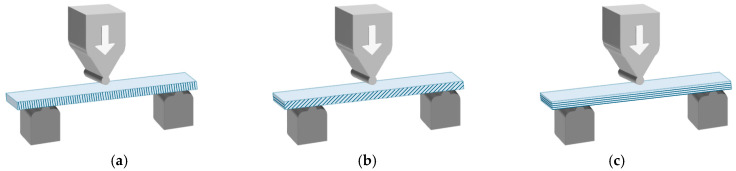
Experimental setup for the three-point bending test of a resin specimen printed at 90° (**a**), 40° (**b**), and 0° (**c**) to the build platform.

Testing was performed using a universal testing machine (ESM 301 Mark-10, Mark-10 Corporation, Copiague, NY, USA) equipped with a 50 N load cell (M5-50, Mark-10, Copiague, NY, USA), at a crosshead speed of 5 mm/min. The test was terminated when the specimen deflection reached 15 mm, according to the protocol of Perea-Lowery et al. [10]. The direction of the applied load in relation to the printed resin layers is illustrated in Figure 3.

The fracture load was recorded in Newtons (N), and the flexural strength (σ) was calculated in megapascals (MPa) using the following formula:

$$\sigma =\frac{3Fl}{2wh^{2}}$$

The flexural modulus (E) was calculated in gigapascals (GPa) using the formula:

$$E=\frac{Fl^{3}}{4wh^{3}d}$$

where F is the fracture load in Newton, l is the distance between the supports in millimeters, w is the width in millimeters, h is the height in millimeters and d is the deflection in millimeters at load F.

### 2.4. Statistical Analysis

#### 2.4.1. Flexural Strength

As the overall distribution of the collected flexural strength data was not normal according to the Shapiro–Wilk test, the use of a three-way Analysis of Variance (ANOVA) with flexural strength as the dependent variable, material type, print angulation, and water storage as factors was precluded. Thereby, two separate statistical analyses were applied to unaged and aged specimens.

#### 2.4.2. Flexural Strength of Unaged Specimens

As the data met the requirements of normality of data distribution (Shapiro–Wilk test) and homogeneity of group variances (Levene test), the two-way ANOVA was applied, with flexural strength as the dependent variable, material type and print angulation as the independent variables. The statistical significance of each factor, as well as of the between-factor interaction, was assessed. The Tukey test was applied for post hoc comparisons as needed.

#### 2.4.3. Flexural Strength of Aged Specimens

The finding that the data distribution was not normal according to the Shapiro–Wilk test ruled out the use of a two-way ANOVA with flexural strength as the dependent variable, material type, and print angulation as factors. Therefore, two distinct one-way ANOVAs had to be applied to the data, separately assessing the statistical significance of the influence of material type and of print angulation. The Tukey test was used for post hoc comparisons as needed.

#### 2.4.4. Flexural Modulus

The same statistical analysis as for the flexural strength data of aged specimens had to be applied to the flexural modulus data of aged and unaged specimens separately.

In all the tests, the level of significance was set at *p* < 0.05. Statistical calculations were handled by the PASW Statistics 18 software (SPSS Inc., Chicago, IL, USA).

## 3. Results

### 3.1. Flexural Strength of Unaged Specimens

Table 2 reports the descriptive statistics of flexural strength measurements in MPa of unaged specimens, along with the outcome of the statistical analysis. The two-way ANOVA disclosed that the type of material was an effective factor for flexural strength per se (*p* < 0.001). Specifically, regardless of the print angulation, CO exhibited a significantly lower flexural strength than CL. Print angulation was also a significant factor per se (*p* < 0.001). Irrespective of the resin type, specimens printed at 40° had the lowest, and those printed vertically had the highest flexural strength. All the differences among print angulations were statistically significant according to the Tukey test (*p* < 0.05).

The between-factor interaction was also statistically significant (*p* < 0.05). The Tukey test revealed that CL specimens printed at 40° had significantly lower flexural strength than those printed horizontally or vertically, which were similar to each other. Also, CO specimens measured the lowest strength values when printed at 40°, while the highest values were recorded by specimens printed vertically. All the differences among print angulations within the CO group were statistically significant. Additionally, it emerged from the Tukey test that CO yielded significantly lower flexural strength than CL when printing was done horizontally or at 40°, while in vertical prints, the difference between the two resins was not statistically significant.

### 3.2. Flexural Strength of Aged Specimens

Table 3 reports the descriptive statistics of flexural strength measurements in MPa of aged specimens, along with the outcome of the statistical analysis. Water storage reduced the flexural strength in all the experimental groups. For CO, the greatest decrease in mean flexural strength occurred for vertically printed specimens (51.18% flexural strength reduction). The flexural strength values of CO remained significantly lower than those of CL and significantly different among print angulations (*p* < 0.05). Among CL specimens, those printed horizontally manifested the greatest reduction in mean flexural strength with water storage (17.92%) and recorded significantly lower values than vertical prints (*p* < 0.05).

### 3.3. Flexural Modulus of Unaged Specimens

Table 4 presents the descriptive statistics of flexural modulus values in GPa of unaged specimens. CO resin exhibited significantly lower flexural modulus than CL resin (*p* < 0.05). For either resin, flexural modulus increased with increasing print angulation, and the differences were statistically significant (*p* < 0.05), except for the CL 0–40° comparison (*p* > 0.05). CO specimens printed horizontally and CL specimens printed vertically had, respectively, the lowest and the highest flexural modulus.

### 3.4. Flexural Modulus of Aged Specimens

Table 5 presents the descriptive statistics of flexural modulus values in GPa of aged specimens. Similarly to flexural strength, flexural modulus was decreased by water storage in all the experimental groups, and the greatest reduction in mean flexural modulus was recorded for CO resin in vertically printed specimens (48.16%), while for CL resin in horizontal prints (19.96%). Also, after ageing, CO specimens exhibited significantly lower flexural modulus than CL specimens (*p* < 0.05). For CL resin, flexural modulus increased significantly with increasing print angulations, and 90° specimens recorded the highest flexural modulus (*p* < 0.05). Conversely, aged CO specimens displayed very low flexural modulus values, which were statistically similar regardless of the print angulation (*p* > 0.05).

## 4. Discussion

The present study findings revealed significant differences in the flexural strength and flexural modulus of occlusal splint resins printed at different orientations. Moreover, water ageing considerably impacted flexural properties. Thereby, both formulated null hypotheses were rejected herein.

Flexural strength refers to the ability of a material to withstand bending under the application of an external force, quantified as the highest stress the material can endure before failure. Additionally, flexural modulus represents the material’s initial resistance to bending under an applied load, wherein a higher modulus indicates a stiffer structure made from the material [30,38]. Flexural strength and flexural modulus are key mechanical properties used to assess the long-term clinical performance of dental resins undergoing masticatory forces [30]. Considering the absence of an ISO standard specifically designated for occlusal splint materials, the present study adopted the ISO 20795-2:2013 standards [36], indicated for orthodontic base polymers used in orthodontics, as had been done in previous studies [8,9,39]. According to these standards, the minimum requirement for flexural strength is 50 MPa, while the threshold for flexural modulus is 1.5 GPa [36]. However, these standards do not specifically apply to the materials object of the study; therefore, the reported values for flexural strength should only be considered as a “safety value”.

Regarding flexural modulus, the threshold value reported in the standard should be treated with caution, since a very high modulus is not the goal, and a more precise range of modulus values should be determined on the basis of the splint function (protective or rehabilitative). Moreover, there are distinct disparities between the chemical compositions of traditionally manufactured acrylic resin splints and additively manufactured occlusal devices, which raise questions about the applicability of such conceived standards and, in turn, challenge the validity of interpreting the previously reported findings and the clinical relevance therein.

It is also noteworthy to highlight the absence of a standardized ageing protocol exclusively indicated for 3D-printed specimens. The employed ageing regimen in the present study involved one month of water storage isothermally at 37 °C [7,10,15,40]. This was based on the evidence that occlusal splints do not undergo thermal fluctuations in the oral cavity, considering they are used by patients during the night and not subjected to dietary components of varying temperatures. Nevertheless, the reported ageing protocols in the literature varied widely, ranging from 50 h at 37 °C [9] to 10 or 14 days [41,42], and up to 60 days [21]. Likewise, variations exist in thermocycling ageing regimens, ranging from thermocycling between 5 °C and 55 °C temperatures for a duration of 1 min per cycle for 10,000 cycles [43,44], to 30 s per cycle for 3860 cycles [45], and up to 80,000 cycles [19]. The lack of standardization in defining precise storage times that simulate the oral environment may contribute to discrepancies in the measured flexural properties.

In the present study, when tested immediately after fabrication, all unaged resin groups exceeded the ISO recommended minimum requirement flexural strength threshold (>50 MPa) [36]. The findings of the CL are within the range of the values reported in previous similar studies. While the observed results are consistent with those stated in a study by Prpic et al. (75.25 MPa) [8] and Simeon et al. (78.86–85.81 MPa) [19], they are higher than those reported by Nakornnoi et al. (35.09 MPa) [46], and considerably lower than those reported by Aretxabaleta et al. (147.7 MPa) [47]. Such dissimilarities in flexural strength values may be explained by the disparities among specimen dimensions. Prpic et al. [8] as well as Simeon et al. [19] indeed employed similar dimensions as the present study (64.0 × 10.0 × 3.3 mm and 64.0 × 11.0 × 4.0 mm, respectively), while Nakornnoi et al. used larger specimens (80 × 10 × 1 mm) [46], and Aretxabaleta et al. [47] tested much smaller specimen geometries (25  ×  2 × 2 mm). Such diversities in load-bearing areas may plausibly have led to differences in stress distribution and flexural strength.

Conversely, to date, no study has investigated the flexural strength of CO, which demonstrated lower flexural strength values compared to CL. Such a difference may be ascribed to variations in the chemical composition of the resins, which play a crucial role in determining their mechanical properties, as the final characteristics of resin-based materials are largely determined by the interactions within the monomer mixture and the characteristics of the resultant polymer network [10,48]. CL resin is composed of bisphenol A dimethacrylate (Bis-GMA) by 50% to 70% in weight, with lesser quantities of urethane dimethacrylate (UDMA, 25–45 wt%). Differently, the CO group was predominantly composed of UDMA, by 55% to 75% in weight. Although UDMA can form hydrogen bonds by virtue of its urethane groups, these interactions are not as strong as those found in Bis-GMA. Consequently, resins with high amounts of UDMA tend to exhibit higher flexibility, along with a superior degree of monomer conversion and greater morphological homogeneity. In contrast, the hydroxyl groups in Bis-GMA, combined with its rigid core, result in extensive hydrogen bonding networks, yielding higher viscosity and greater stiffness [49,50]. Moreover, the recorded flexural strength data for both resin materials were considerably lower than those reported by the manufacturer (CL = 84 MPa, CO = 21 MPa) [51,52]. It should however be noted that the manufacturer employed the four-point bending test in line with the ASTM D790-15 standards procedure B [53], whereas in the present study, the three-point bending test was conducted according to the ISO 20795-2:2013 standards [36]. Previous studies have confirmed that higher flexural strength values are obtained from three-point bending tests by virtue of the concentrated applied force at one contact point, whereas in the four-point bending test, the applied force is distributed over a broader surface area, thus fostering more uniform stress distribution and lower flexural strength values [54,55]. Nonetheless, for layer-wise 3D-printed structures, the three-point bending apparatus may be advantageous in accurately quantifying the flexural strength values because it can detect weak interlayer networks—which is largely beneficial in the context of this research—while the four-point bending apparatus has a tendency to “average out” these effects over a larger surface area.

Despite the introduction into the market of several resin formulations meant for occlusal splint fabrication, there has been limited research assessing their long-term mechanical properties [11,13,15,26,27]. Additionally, the majority of previous comparisons have been drawn between conventional manufacturing methods and 3D printing [5,7,8,9,41,42,56,57]. However, the increased adoption of 3D printing necessitates a thorough evaluation of the impact of printing parameters. Indeed, the orientation of objects on the build platform influences fabrication accuracy, printing time, and post-processing requirements by altering the number and dimensions of overlapping layers [20]. In vertical printing (90°), specimens are built up perpendicular to the printing platform, and each layer comprises a reduced surface area. On the other hand, in horizontal printing (0°), specimens are layered parallel to the printing platform, with each layer displaying a larger surface area. Furthermore, 90° orientation permits the accommodation of a greater number of objects on the build platform, thus reducing resin consumption and the necessity for extensive support structures, which, in turn, streamlines the finishing process. Conversely, 0° orientation results in fewer layers, thereby accelerating the printing process [19]. For these reasons, the present study evaluated specimens printed at 0°, 90°, and at the manufacturer-recommended 40° angulation. While the printing orientation considerably influenced the flexural properties of both resin materials to varying degrees, the specimens printed perpendicular to the printing platform consistently exhibited superior flexural strength and modulus. This observation has been corroborated in the literature, wherein additively manufactured occlusal splints exhibited the highest flexural strengths when printed vertically [19,21]. Therefore, it can be inferred that when printed resin layers align parallel to the compressive force, an anisotropic structure is created that mitigates tension-induced fracture. Moreover, vertical orientation produces minimal resin build-up due to gravity, thereby yielding greater homogeneity and coherence between printed resin layers [58]. Conversely, the 40° printing orientation yielded the lowest flexural strengths for both resins, which may be attributed to the positioning of the printed layers relative to the applied load or to inadequate layer adhesion. Thus, when force is exerted at an angle that is not perpendicular to the layers (Figure 3b), it may lead to increased deflection and reduced resistance to bending among the external resin layers. However, although the observed reduction of approximately 4 MPa at 40° is statistically significant, it is still considered modest and remains within ISO requirements.

In the present study, the flexural modulus parameter of unaged specimens was also significantly affected by material type and printing orientation, with measured values falling below the ISO 20795-2:2013 requirement (<1.5 GPa) [36], particularly for the CO resin. The significantly lower flexural modulus of CO is in line with the manufacturer’s aim to produce a material with greater flexibility and comfort [52]. Concerning CL, the flexural modulus values recorded in the present study were lower than those declared by the manufacturer (2.3 GPa) [51], and also inferior to those reported by Simeon et al. (1.94–2.09 GPa) [19] and Nakornnoi et al. (1.25–1.55 GPa) [46]. This may be justified by the dissimilar flexural test apparatuses (three-point bending vs. four-point bending) [51], varying specimen dimensions [46], or the parafilm taping of the support pins to prevent specimen sliding and eliminate unwanted friction at the supports [19]. Additionally, specimens printed at 0° demonstrated the lowest flexural modulus for either tested resin, indicating greater stiffness therein. This may be explained by the unfavorable resin build up when printing parallel to the platform, which may cause imbalanced polymerization and adhesion among printed resin layers [58]. While the observed effect of printing angulation on flexural strength and modulus is relatively moderate, it still impacts the overall load-bearing capacity of the printed material. Clinically, these findings indicate perpendicular printing orientation as the most effective for occlusal device applications [41].

It was reported by Neoh et al. [43] that after water immersion, aged CL specimens presented increased surface irregularities and roughness, compared to thermoplastic sheet resins. In contrast, milled occlusal devices offered superior mechanical properties [8], as well as fewer porosities and a higher degree of polymerization [9,59]. Berli et al. reported that two out of three 3D-printed materials absorbed twice as much water as pressed and milled counterparts, indicating higher porosity in the former, a factor that may further compromise their mechanical performance [9]. Exposure to water has multiple effects on the properties of resins, encompassing plasticization and softening of the matrix, along with the elution of unreacted monomers and small oligomers into the oral cavity. Additionally, water sorption results in an overall expansion of the device [33]. In the present investigation, a notable decrease in flexural strength was observed for both CL and CO after one month of water storage. These findings align with those reported by similar studies [9,13,15,26,27], where artificial ageing significantly deteriorated the flexural strength of 3D-printed splint materials. In addition, Xu et al. [17] reported that prolonged washing of occlusal splints adversely affected their flexural strength. Nonetheless, among CL specimens, no differences were observed between printing orientations, and acceptable flexural strength values were maintained after ageing. In contrast, aged CO specimens printed at 40° had their flexural strength reduced by approximately half after water storage, with values falling below the threshold of acceptability set by ISO standards [36]. This occurrence may be ascribed to the hydrophilic nature of urethane linkages in UDMA, which may have further facilitated water penetration in CO [48]. The flexural strength outcome of CO raises concerns about its long-term clinical performance when subjected to heavy masticatory forces.

Likewise, water storage also led to a decrease in flexural modulus in both resin groups (<0.5 GPa), with a similar trend observed among printing orientations and a more pronounced reduction detected in CO compared to CL. Despite the lack of consensus and the complex informed decisions regarding splint material selections [9], in clinical practice, the splint materials that display greater flexibility under masticatory forces might lead to premature wear of the occlusal surfaces and are thereby considered less favorable for the treatment of TMJ disorders, where firmer splints are often preferred to ensure timely symptom relief [39].

A limitation of the present study can be considered that the investigated resins herein allow a 100 µm printed layer thickness, while other printable resins offer the possibility of printing layers with lower thickness, such as 50 µm. Therefore, the effect of layer thickness on flexural properties cannot be concluded only based on the present findings. Reducing layer thickness increases the number of layers and interfaces, which could amplify the observed differences among printing orientations. Previous research revealed that decreasing layer thickness can enhance the strength of printed resin prostheses [15,41,60,61]. Another limitation of the study is that polishing has not been performed, even if it has been reported in the literature that polishing can increase the resistance of the specimens to artificial aging for Dental LT [29], polishing was not performed in the present study to avoid biasing the results, as the effect of polishing on Comfort Resin is not known. Further studies could clarify this aspect. Furthermore, a comprehensive understanding of the failure modes in the present study can be obtained through the addition of microscopic characterization and fractographic analysis of the resultant fracture patterns. Another limitation is that the implemented ageing protocol of 30-day continuous water storage does not fully replicate the clinical conditions. Thus, additional studies are needed to evaluate the long-term performance of the available materials under simulated oral conditions, such as dynamic loading or, even more relevantly, in the clinical setting. A deeper understanding of the impact of experimental testing variables will aid in optimizing material selection and printing parameters, aiming at enhancing the durability and clinical performance of occlusal splints.

## 5. Conclusions

1. Dental LT Comfort resin exhibited significantly lower flexural strength and modulus compared to Dental LT Clear v2 resin.
2. 3D printed occlusal splint resin specimens printed at 90° consistently demonstrated superior flexural strength and modulus.
3. For both resins, printing at 40° to the build platform, as recommended by the manufacturer, resulted in significantly lower flexural strength.
4. After water storage, the flexural strength and modulus decreased for both resins, with Dental LT Comfort experiencing up to 52% loss in flexural strength and expressing values that fell below the ISO threshold for clinical acceptability.

## Figures and Tables

**Table 1:** Description of characteristics and compositions of the investigated dental resins.

Material (Manufacturer)	Manufacturing Method	Abbreviation	System (Manufacturer)	Composition (wt%)
Dental LT Clear v2 (Formlabs, Somerville, MA, USA)	Low force SLA printing	CL	Form 3B (Formlabs)	Bisphenol A dimethacrylate (50–70%) Urethane dimethacrylate (25–45%) Methacrylate Monomer(s) (7–10%) Photoinitiator(s) (<2%)
Dental LT Comfort (Formlabs, Somerville, MA, USA)	Low force SLA printing	CO	Form 3B (Formlabs)	Urethane dimethacrylate (55–75%) PEG dimethacrylate (15–25%) Methacrylate Monomer (10–20%) Initiator (<1%)

**Table 2:** Descriptive statistics of the flexural strength values (MPa) of unaged specimens.

Material	Print Angulation	N	Mean ± SD
Dental LT Clear Resin (CL)	0°	10	72.65 ± 1.83 ^Aa^
	40°	10	68.31 ± 1.46 ^Ba^
	90°	10	72.74 ± 1.02 ^Aa^
	Total	30	71.23 ± 2.54
Dental LT Comfort Resin (CO)	0°	10	66.89 ± 2.08 ^Bb^
	40°	10	62.71 ± 1.21 ^Cb^
	90°	10	71.47 ± 1.26 ^Aa^
	Total	30	67.03 ± 3.94
Total print angulations	0°	20	69.77 ± 3.51 ^&^
	40°	20	65.51 ± 3.15 ^#^
	90°	20	72.11 ± 1.29 *

Different superscript uppercase letters represent significant differences between print angulations within the same material. Different superscript lowercase letters represent significant differences between materials within the same printing angulation. Different superscript symbols represent significant differences between print angulations regardless of material type (*p* < 0.05).

**Table 3:** Descriptive statistics of the flexural strength values in MPa of aged specimens.

Material	Print Angulation	N	Mean	Std. Deviation
Dental LT Clear Resin (CL) *	0° ^B^	10	59.63	2.39
	40° ^AB^	10	61.55	0.79
	90° ^A^	10	62.91	2.19
Dental LT Comfort Resin (CO) ^§^	0° ^C^	10	42.14	2.077
	40° ^E^	10	30.61	1.57
	90° ^D^	10	34.08	0.98

Different symbols label the statistically significant difference between the two materials, per se. Different superscript letters label all the other statistically significant between-group differences (*p* < 0.05).

**Table 4:** Descriptive statistics of flexural modulus values in GPa of unaged specimens.

Material	Print Angulation	N	Mean	Std. Deviation
Dental LT Clear Resin (CL) *	0° ^B^	10	0.541	0.017
	40° ^B^	10	0.545	0.015
	90° ^A^	10	0.582	0.008
Dental LT Comfort Resin (CO) ^§^	0° ^E^	10	0.159	0.006
	40° ^D^	10	0.224	0.007
	90° ^C^	10	0.245	0.005

Different symbols label the statistically significant difference between the two materials, per se. Different superscript letters label all the other statistically significant between-group differences (*p* < 0.05).

**Table 5:** Descriptive statistics of flexural modulus values in GPa of aged specimens.

Material	Print Angulation	N	Mean	Std. Deviation
Dental LT Clear Resin (CL) *	0° ^C^	10	0.43	0.018
	40° ^B^	10	0.48	0.006
	90° ^A^	10	0.49	0.016
Dental LT Comfort Resin (CO) ^§^	0° ^D^	10	0.11	0.005
	40° ^D^	10	0.11	0.006
	90° ^D^	10	0.12	0.003

Different symbols label the statistically significant difference between the two materials, per se. Different superscript letters label all the other statistically significant between-group differences (*p* < 0.05).

## Data Availability

The original contributions presented in this study are included in the article. Further inquiries can be directed to the corresponding author.
